# Early experience with the Aristotle Colossus 0.035’ macrowire for mechanical thrombectomy in 30 consecutive cases

**DOI:** 10.1177/15910199241299480

**Published:** 2024-12-10

**Authors:** David A Zarrin, Fahad J Laghari, Jessica K Campos, Benjamen M Meyer, Muhammad W Khan, Jonathan Collard de Beaufort, Gizal Amin, Narlin B Beaty, Matthew T Bender, Shuichi Suzuki, Geoffrey P Colby, Alexander L Coon

**Affiliations:** 1 David Geffen School of Medicine, University of California Los Angeles, Los Angeles, CA, USA; 2 Carondelet Neurological Institute, St. Joseph's Hospital, Tucson, AZ, USA; 3 Department of Neurological Surgery, 8788 University of California Irvine, Orange, CA, USA; 4 College of Medicine, University of Arizona, Tucson, AZ, USA; 5 2029 Syracuse University, Syracuse, NY, USA; 6 Department of Neurosurgery, Florida State University, Tallahassee Memorial Hospital, Tallahassee, FL, USA; 7 Department of Neurosurgery, 6927 University of Rochester, Rochester, NY, USA; 8 Department of Neurosurgery, University of California Los Angeles, Los Angeles, CA, USA

**Keywords:** Guidewire, mechanical thrombectomy, acute stroke

## Abstract

**Introduction:**

Recent literature continues to demonstrate the successful role of large-bore aspiration catheters in thrombus ingestion during mechanical thrombectomy. However catheter-to-microwire step-off and distal navigation are ongoing challenges in thrombectomy. A new to market 0.035’ macrowire (Aristotle 35 Colossus Guidewire, Scientia Vascular, West Vale City, UT) may address such challenges. We report here our early experience in 30 mechanical thrombectomy cases.

**Materials and Methods:**

We analyzed a prospectively maintained database of the senior authors to identify cases utilizing a 0.035’ macrowire with 0.035’ aspiration catheters for mechanical thrombectomy.

**Results:**

Thirty consecutive cases were identified. Seventeen (57%) patients were female with an average age of 75.3 ± 2.2 years (range 55–97). Average presenting NIHSS was 13.0 ± 1.7. Thrombus locations included 7% (*n* = 2) in the cervical ICA, 47% (*n* = 14) in the M1, 43% (*n* = 13) in the M2, and 3% (*n* = 1) in the P1. An 088’ ID aspiration catheter was navigated to at least the M1 segment in all anterior circulation cases and the basilar in the posterior circulation case. The 0.035’ macrowire was placed proximal to the occlusion in all cases allowing coaxial 035’ and 071’ catheter aspiration passes. TICI 2C/3 was achieved in 87% of cases (*n* = 26) and TICI 2B in the remaining cases. There were no wire-related perforations or vessel dissections.

**Conclusion:**

The Colossus 0.035’ macrowire may offer advantages over its smaller counterparts by reducing ledge effect and the need to cross the thrombus. Further comparative studies against currently available microwires in various anatomies are warranted.

## Introduction

The efficacy of mechanical thrombectomy hinges on the ability to navigate the cerebral vasculature with precision and safety, making the choice of guidewire a critical component of the intervention strategy.^1^ The triaxial system, encompassing a guide catheter, intermediate catheter, and microcatheter tracking over a guidewire, epitomizes the current standard in achieving both proximal support and distal flexibility essential for successful clot engagement and removal.^2^ However, the intricacies of vascular anatomy has demanded continuous innovation in device design to optimize the outcomes in mechanical thrombectomy.

Recent advances have seen the development of guidewires with enhanced mechanical properties, aimed at overcoming challenges posed by tortuous pathways and the need for effective thrombus management.^3^ The Aristotle 35 Colossus Guidewire represents such an innovation, designed to address the limitations of existing systems in navigating through small caliber distal vasculature. The traditional concern with microwires—namely, the ledge gap or step-off between the microwire and the microcatheter—can significantly impact the success of the procedure. Larger diameters of microwires have the potential to mitigate this issue by closely matching the inner diameter of the catheter system, thus reducing navigation impediments and the risk of distal embolization caused by clot disruption. However, this must be achieved while maintaining the performance benefits of traditional smaller microwires, such as torqueability and softness. Previous comparisons between 0.014” guidewires and larger 0.018” and 0.024” wires have shown a reduction in procedure time and an increase in first-pass success rates.^4^ These findings highlight the potential benefits of larger diameter guidewires in mechanical thrombectomy, setting the stage for the introduction of the Colossus 0.035” macrowire.

In this study, we review our experience with the Colossus 0.035’ macrowire across 30 consecutive mechanical thrombectomy cases. Our objective was to evaluate its performance in navigating the cerebrovascular anatomy and to assess its impact on procedural efficiency and patient outcomes. We aim to offer insights into the practical application of this novel macrowire and its place within the evolving landscape of thrombectomy techniques.

## Methods

### Patient selection

A prospectively maintained, IRB-approved multi-center institutional database of the senior authors was retrospectively reviewed to identify all mechanical thrombectomy cases in which the Colossus Guidewire was used.

### Procedural details

In all cases, an 8-French short sheath was utilized to gain access to the femoral artery using Seldinger technique and maintained under continuous flush. Under real-time fluoroscopy, the Colossus macrowire was introduced and navigated into the internal carotid artery. An 0.088’ distal access catheter (Zoom 88) which was navigated triaxially over smaller caliber distal access catheters (Zoom 71 and Zoom 35) and the 0.035’ Colossus macrowire. The 0.088’ catheter was positioned just proximal to the clot. The Penumbra Aspiration Pump or Zoom Pump and Zoom POD were used to apply vacuum through all the catheters and aspirate the clot. For cases in which a stentriever was used, either a Zoom 71 or 55 with Zoom 35 and a macrowire system were advanced through Zoom 88 to interface with the thrombus. In cases of stentriever usage, the Zoom 35 was removed and a 0.021’ microcatheter was utilized to deploy a stentriever. The Zoom 88 was advanced over the stentriever to the clot, before removing the stentriever under vacuum aspiration through the Zoom 88. Follow-up angiography was performed to confirm revascularization before concluding the case.

### Data collection

The electronic medical record of all qualifying cases was reviewed for the following information: age, sex, comorbidities, thrombolytic administration, thrombus location, and presenting National Institutes of Health Stroke Scale (NIHSS). Anterior circulation mechanical thrombectomy was characterized by the following on angiography: cervical ICA tortuosity (corkscrew loop or 90° turn), petrous grading (hockey stick, some recurve, or question mark) and cavernous ICA tortuosity (Ia, Ib, II, III, or IV).^5^ Case characteristics, including catheter system used, total fluoroscopy time, procedural contrast (cc/mL), and radiation dose (mGy) were collected. Immediate procedural outcomes, including modified thrombolysis in cerebral infarction (mTICI) reperfusion grade and the number of passes, were obtained. Each case was scrutinized for vasospasm and catheter or macrowire-related vessel injury.

### Outcomes

Clinical outcomes extracted for each case include immediate pre- and post-operative deficit and post-operative re-infarct. Procedural outcomes included degree of recanalization, number of passes to achieve recanalization, the distal most position of the Colossus macrowire, clot traversal or not, intraoperative complications such as vasospasm or vessel dissection. Technical success was defined as successful positioning of the Colossus macrowire at the level of the thrombus without traversal.

### Statistical analysis

All statistical analyses were performed using Minitab 21.4.0 statistical software (Minitab Inc., State College, PA). Two-sample *t*-tests and Chi-squared tests were performed to assess statistical significance between groups for continuous data and categorical data, respectively.

## Results

From April 2023 to October 2023, thirty consecutive thrombectomy cases using an 0.035’ macrowire were reviewed, with 30 cases (100%) achieving technical success. Demographic, thrombus, and anatomy characteristics are presented in Table 1. Just over half (*n* = 17, 57%) of patients were female and the average age was 75.3 ± 2.2 years. The average presenting NIHSS was 13.0 ± 1.7, with 53% (*n* = 16) of thrombi located on the left side. Thrombolytics were used infrequently. Most thrombi (47%, *n* = 14) were in the MCA-M1 segment, followed by 43% (*n* = 13) in the M2, two cases (7%) in the cervical ICA, and one case in the PCA-P1 segment. 14 (47%), 6 (20%), and 9 (30%) of the petrous ICA segments were scored as a hockey stick, some recurve, or question mark shape, respectively. The most common type of cavernous grade was Ia (*n* = 11, 37%), followed by IV (*n* = 7, 23%), Ib (*n* = 7, 23%), III (*n* = 3, 10%), and II (*n* = 1, 3%). There was one (3%) case of non-flow limiting vasospasm at the location of the thrombus that was managed with 10 mg of intra-arterial verapamil.

**Table 1.** Baseline characteristics, anatomy, and thrombus location of the study patients.

	Number/Range	%/SE
*Demographics*		
Total cases	30	
Age (years)	75.3	2.2
Female sex	17	57%
Thrombolytics	7	23%
NIHSS score on presentation	13	1.71
Comorbidities		
Hypertension	13	43%
Atrial fibrillation	5	17%
Hyperlipidemia	5	17%
Type II diabetes	3	10%
Cancer	2	7.0%
Patent foramen ovale	1	3.0%
Smoking history	1	4.0%
*Thrombus location*		
Laterality		
Left	16	53%
Right	14	47%
Branch		
Cervical ICA	2	7%
M1	14	47%
M2	13	43%
P1	1	3%
*Anatomy*		
Presence of cervical tortuosity	3	10%
Petrous grade		
Hockey stick	14	47%
Some recurve	6	20%
Question mark	9	30%
Cavernous Grade		
Ia	11	37%
Ib	7	23%
II	1	3%
III	3	10%
IV	7	23%

NIHSS indicates National Institutes of Health Stroke Scale and SE indicates standard error.

**Figure 1.**
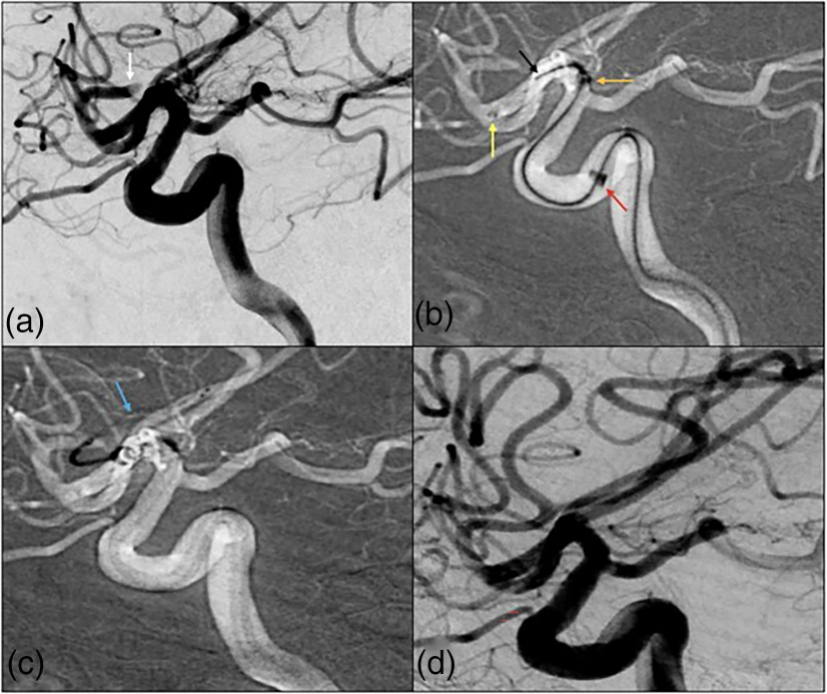
(a) A patient in their 80s presents with an NIHSS of 17 with a thrombus in the left superior M2 division (white). (b) 035’ (yellow), 071’ (orange), and 088’ (red) aspiration catheters over the Colossus 35 (black) micro-guidewire in a Type 4 cavernous ICA near the level of the thrombus without crossing it. (c) Vector was again achieved in the M2 with a 014’ Synchro 2 guidewire and 021’ microcatheter, the guidewire was removed, and a 3 × 32 stentriever (blue) was deployed. (d) TICI 3 recanalization was achieved in one pass (colors listed as distal to proximal arrows within figure).

**Figure 2.**
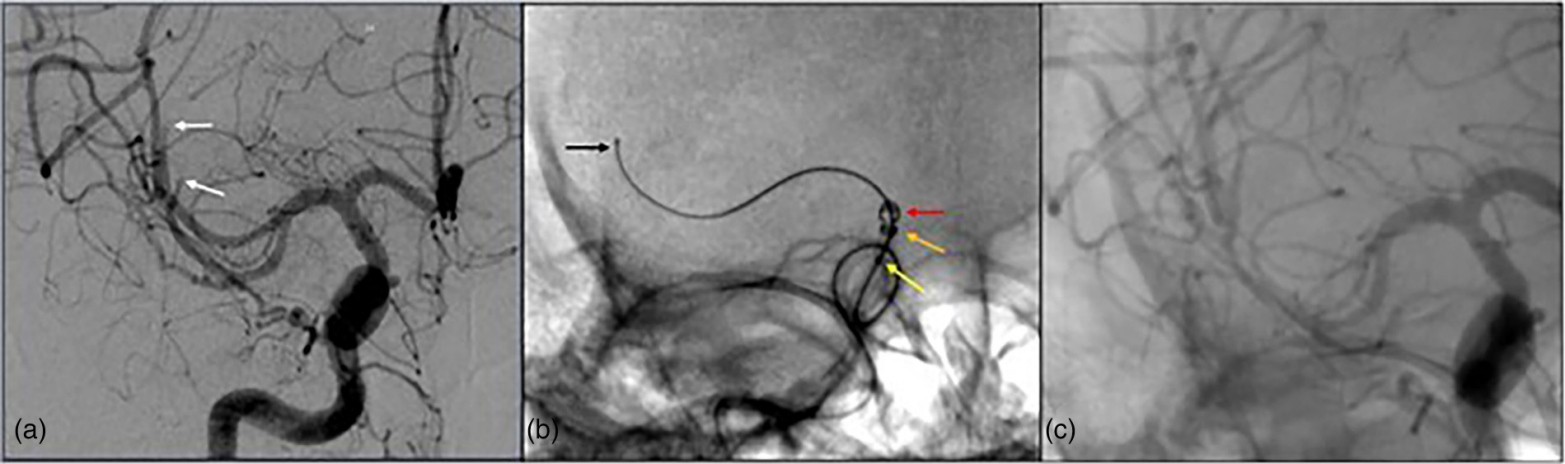
(a) A patient in their 80s presents with an NIHSS of 17 with a thrombus in the left superior M2 division (white). (b) 035’ (yellow), 071’ (orange), and 088’ (red) aspiration catheters over the Colossus 35 (black) micro-guidewire in a Type 4 cavernous ICA near the level of the thrombus without crossing it. (c) Vector was again achieved in the M2 with a 014’ Synchro 2 guidewire and 021’ microcatheter, the guidewire was removed, and a 3 × 32 stentriever (blue) was deployed. (d) TICI 3 recanalization was achieved in one pass (colors listed as distal to proximal arrows within figure).

**Figure 3.**
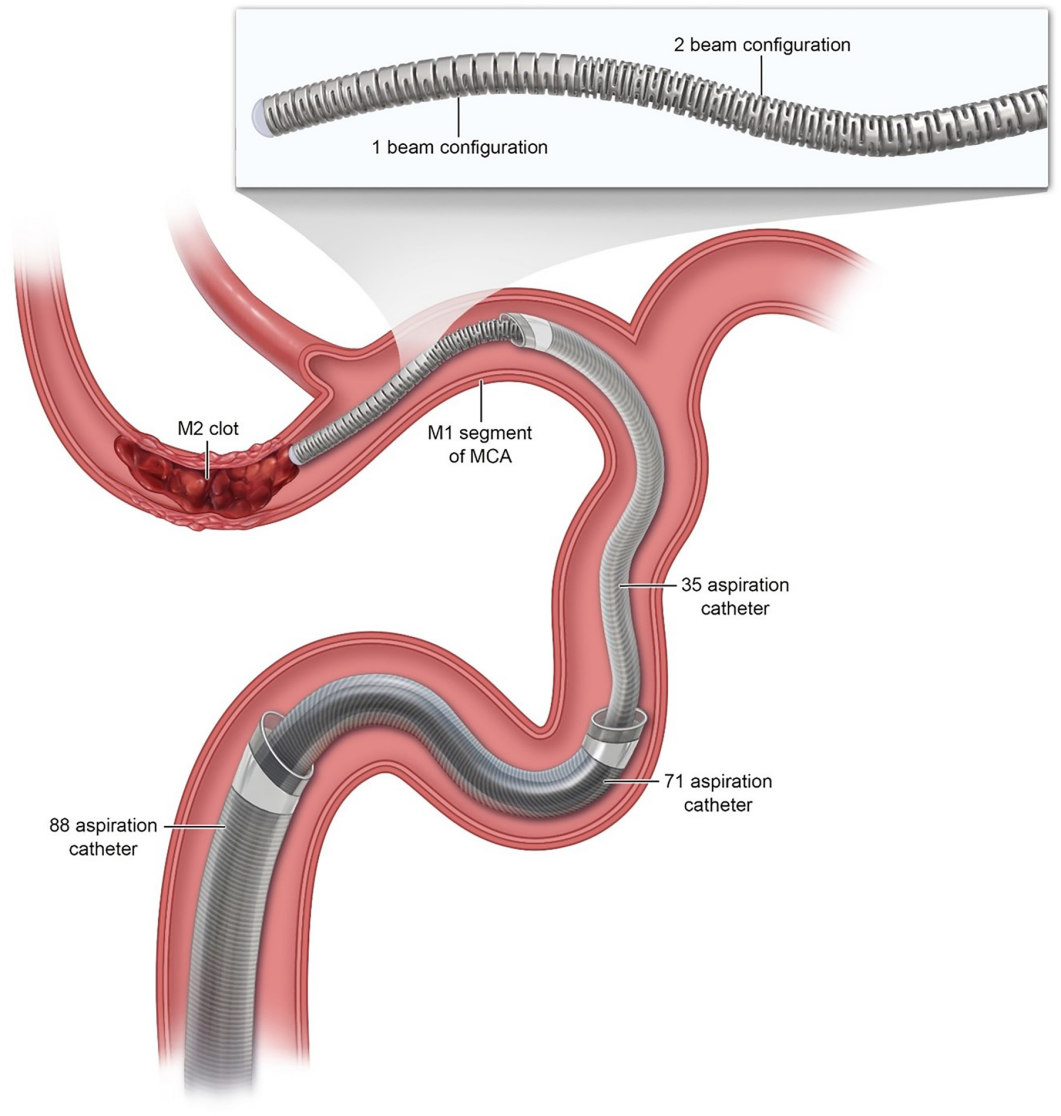
Illustration of the typical mechanical thrombectomy access system configuration utilized in our approach, demonstrating 088’ and 071’ aspiration catheters positioned in the ICA, an 035’ aspiration catheter in the proximal M1 segment of the MCA, and the Colossus 035’ macrowire in its distal-most position, proximal to the M2-segment clot. The zoomed panel demonstrates the novel graduated peripheral cut pattern of Colossus, which simultaneously allows for stiffer and more supportive proximal segments and more torquable distal segments of the microwire.

The most common catheter systems used include Zoom88/55 and Zoom88/71/35 (see Table 2), both with Trak 21 and a stentriever (see Figure 1). Colossus 35 was most commonly navigated to the M1 (47%, *n* = 14) segment, and clot transversal was not required in any of the cases (see Figure 2). An illustration of a typical access system utilized in our approach is shown in Figure 3. The average fluoroscopy time was 22.1 ± 2.4 minutes, radiation exposure was 697 ± 83 mGy, and contrast was 25.1 ± 2.7 mL. First-pass mTICI ≥2C recanalization was achieved in 47% (*n* = 14) cases. mTICI ≥2B recanalization was achieved in all cases, and the average number of passes was 1.7 ± 0.2. There were no macrowire-related perforations or vessel dissections.

**Table 2.** Procedural details and outcomes.

	Number/Range	%/SE
*Systems Used*		
Zoom 88/Zoom 55/Trak 21/Trevo 4 × 28	5	17%
Zoom 88/Zoom 71/Zoom 35/Trak 21/Trevo 4 × 28	4	13%
Zoom 88/Zoom 71/Zoom 35	4	13%
Zoom 88/Zoom 55/Trak 21/Trevo 4 × 41	2	7%
Zoom 88/Zoom 55/Trak 21/Trevo 3 × 32	2	7%
Zoom 88/Zoom 71/Zoom 35/Trak 21/Trevo 3 × 32	2	7%
Zoom 88/Zoom 71/Zoom 35/Phenom 21/Trevo 3 × 32	1	3%
Zoom 88/Zoom 55/Phenom 21/Tiger 017	1	3%
Zoom 88/Phenom 21/Trevo 3 × 32	1	3%
Zoom 88/Zoom 71/Zoom 35/SL-10/Trevo 3 × 32	1	3%
Zoom 88/Zoom 55/Trak 21/Tiger 013	1	3%
Zoom 88/Zoom 71/Zoom 35/Trak 21/Tiger 017	1	3%
Zoom 88/Zoom 55/Duo 157/Tiger 013	1	3%
Zoom 88/Zoom 71/Zoom 35/XT-27/Trevo 4 × 28	1	3%
Zoom 88/Zoom 71/Zoom 35/Embotrap 6.5 × 45	1	3%
Zoom 88/Zoom 55/Trak 21/Tiger 017	1	3%
Zoom 88/Zoom 71/Zoom 55/Trak 21/Trevo 4 × 41	1	3%
*Case Characteristics*		
Colossus 35 positioning		
Cervical ICA	2	7%
M1	14	47%
M2	13	43%
P1	1	3%
Clot transversal	0	0%
Fluoroscopy time (minutes)	22.1	2.4
Radiation exposure (mGy)	697	82.7
Contrast amount (mL)	25.1	2.7
*Procedural outcomes*		
TICI 2C/3	26	87%
TICI 2B	4	13%
First-pass efficacy	16	53%
Average number of passes	1.7	0.17
Vasospasm	1	3%
Wire-related perforations	0	0%
Technical success	30	100%

mTICI indicates modified thrombolysis in cerebral infarction reperfusion grade.

## Discussion

The integration of the 0.035’ macrowire underscores a key advancement in mechanical thrombectomy, particularly in accessing and managing distal clots. Traditionally, achieving thrombectomy catheter placement proximal to distal clots often necessitated crossing the thrombus with the microwire, introducing risks of clot disintegration and potential distal embolization. Our findings suggest that the use of this macrowire allows the thrombectomy catheter to approach the clot directly without the necessity for clot traversal with the wire, aligning with the principles of the direct aspiration first pass technique (ADAPT) and other prior literature.^6,7^ This method allows for a safer initial attempt at clot aspiration without the need for clot traversal, with microwires introduced later only if a stentriever becomes necessary. This capability undoubtedly enhances procedural efficacy and safety.

We believe that the larger and softer Colossus macrowire can be safely manipulated within cerebrovascular anatomy in the context of several unique mechanical features of the wire and appropriate technique-of-use. We found the wire to be surprisingly torquable in its distal segments despite its proximal stiffness, possibly owing to its distal peripheral cutting pattern and exceptionally soft, atraumatic tip. ‘The tip of the Colossus wire is shapeable and can be used both with its natural J-shape or a slight hockey stick shape for vessel selection. In our experience, both configurations were successful, providing flexibility depending on the case. The typical setup in our practice involves 0.035” Colossus macrowire in an 0.035’ thrombectomy catheter which is coaxially placed within another 0.071’ thrombectomy catheter and 0.088’ guide catheter. Because the outer diameter of Colossus approximates the inner diameter of the 0.035’ catheter, there is effectively a zero step-off ledge. We felt this feature resulted in reduced friction with endothelial lining during catheter tracking, potentially minimizing vessel wall/endothelial abrasion and the concomitant micro-platelet aggregation that can result in distal embolism.

Our experience indicates that the wire's graduated mechanical stiffness does not compromise the stability of the access system, even without the need for clot passage. This aspect is crucial, given that previous systems relied on clot traversal to maintain access system stability, which increased the risk of iatrogenic distal emboli and infarcts. We found that the 0.035” macrowire's presence within 0.035” and 0.071” catheters adds stability, despite the softness of the wire; its fullness within these catheters provides substantial support when bringing 0.071” and 0.088” catheters into thrombectomy capable positions. It is likely feasible that this macrowire could also be used directly with larger 0.055” or 0.071” thrombectomy catheters for tracking them. This is a routine practice with super soft, non-distal PTFE-lined thrombectomy catheters such as SOFIA (soft torqueable catheter optimized for intracranial access; MicroVention, Aliso Viejo, CA). This point merits further investigation.

The outcomes observed from the use of this macrowire in our series of mechanical thrombectomy procedures—highlighted by favorable modified TICI score distribution, low complication rates, and a high first-pass rate—speak to its efficacy and safety in real-world practice. Although these results could be replicated with other systems, wires, and arrangements, the ease of use of this particular wire enhances procedural efficiency. The enhanced efficiency from using a larger wire, without compromising safety, represents an important advancement as a tool for thrombectomy. Our results are indicative of the macrowire's potential to improve procedural outcomes and patient safety.

While our results are promising, there are limitations. These may include navigational challenges in highly tortuous anatomy or specific scenarios where smaller wires could offer superior flexibility. Future studies, ideally involving larger sample sizes and comparative designs, are needed to further explore these aspects, as well as the wire's efficacy and safety in a wider range of clinical settings.

## Conclusion

The Colossus 0.035’ macrowire represents a significant advancement in the field of mechanical thrombectomy. Its ability to facilitate close positioning to distal clots without requiring traversal, combined with its zero step-off system and atraumatic design, provides a substantial performance advantage over smaller micro-wires during mechanical thrombectomy procedures.
